# Final-year medical students’ self-assessment of facets of competence for beginning residents

**DOI:** 10.1186/s12909-021-03039-2

**Published:** 2022-02-07

**Authors:** Lisa Bußenius, Sigrid Harendza, Hendrik van den Bussche, Susan Selch

**Affiliations:** 1 Department of Biochemistry and Molecular Cell Biology, Center for Experimental Medicine, University Medical Center Hamburg-Eppendorf, Hamburg, Germany; 2 III. Department of Internal Medicine, University Medical Center Hamburg-Eppendorf, Hamburg, Germany; 3 Department of General Practice and Primary Care, University Medical Center Hamburg-Eppendorf, Hamburg, Germany

**Keywords:** Competences, Final-year medical students, Postgraduate medical education, Self-assessment, Undergraduate medical education

## Abstract

**Background:**

Final-year undergraduate medical students often do not feel well prepared for their start of residency training. Self-assessment of competences is important so that medical trainees can take responsibility for their learning. In this study, we investigated how final-year medical students self-assessed their competences as they neared their transition to postgraduate training. The aim was to identify areas for improvement in undergraduate training.

**Methods:**

In the academic year 2019/2020, a national online survey was sent to final-year undergraduate medical students via their respective medical schools. The survey included ten facets of competence (FOC) most relevant for beginning residents. The participants were asked to self-assess their competence for each FOC on a 5-point Likert scale (1: strongly disagree to 5: strongly agree). We established an order of self-assessed FOC performance by means and calculated paired *t*-tests. Gender differences were assessed with independent *t*-tests.

**Results:**

A total of 1083 students from 35 medical schools completed the questionnaire. Mean age was 27.2 ± 3.1 years and 65.8% were female. Students rated their performance highest in the FOCs ‘Teamwork and collegiality’ and ‘Empathy and openness’ (97.1 and 95.0% ‘strongly agree’ or ‘agree’, respectively) and lowest in ‘Verbal communication with colleagues and supervisors’ and ‘Scientifically and empirically grounded method of working’ (22.8 and 40.2% ‘strongly disagree’, ‘disagree’, or ‘neither agree nor disagree’, respectively). Women rated their performance of ‘Teamwork and collegiality’, ‘Empathy and openness’, and ‘Knowing and maintaining own personal bounds and possibilities’ significantly higher than men did (Cohen’s *d* > .2), while men showed higher self-assessed performance in ‘Scientifically and empirically grounded method of working’ than women (Cohen’s *d* = .38). The FOCs ‘Responsibility’, ‘Knowing and maintaining own personal bounds and possibilities’, ‘Structure, work planning, and priorities’, ‘Coping with mistakes’, and ‘Scientifically and empirically grounded method of working’ revealed lower self-assessed performance than the order of FOC relevance established by physicians for beginning residents.

**Conclusions:**

The differences between the level of students’ self-assessed FOC performance and physicians’ ranking of FOC relevance revealed areas for improvement in undergraduate medical education related to health system sciences. Final-year students might benefit from additional or better training in management skills, professionalism, and evidence-based medicine. Surveys of self-assessed competences may be useful to monitor competence development during undergraduate training.

**Supplementary Information:**

The online version contains supplementary material available at 10.1186/s12909-021-03039-2.

## Background

Medical curricula increasingly develop towards achievement of competence [1, 2] and are based on competence frameworks for postgraduate or undergraduate medical education [3, 4]. Such frameworks are helpful to assess readiness for residency [5, 6] and ultimately medical practice [7]. A prior study has empirically proposed a framework of facets of competence (FOCs) which provide a basis for assessing whether or not graduates are ready for clinical practice [8]. This framework has been used in medical education research [5, 6]. Twenty-five FOCs relevant for beginning residents were identified [8] and ranked using a Delphi process by experienced Dutch and German physicians with respect to their importance for beginning residents [9, 10]. In an additional study, faculty and undergraduate medical students agreed on the prioritization of eight of the top ten FOCs most important for the start of residency [11] but some gender differences were also identified [10]. Assessing these FOCs during a simulated first day of residency suggested gaps in performance in which the efficacy of undergraduate training did not match the community’s needs assessment for residency [12].

It is important that medical graduates can self-assess their competence so that they can take responsibility for lifelong learning as physicians [13, 14]. Self-assessment is an important element in medical education to develop clinical competence [15]. The ability to accurately assess one’s own competence – and especially its limits – is also vital in patient care [16]. Additionally, in combination with feedback from faculty, medical trainees’ self-assessment provides a valuable learning tool fostering reflection [16]. Self-assessed competence is important for investigating medical students’ preparedness for clinical practice [17] and affects the transition from medical school to residency [18, 19]. However, medical students’ self-assessment is not always accurate, as both under- and overestimation of competence has been found [15].

Since a gap between trainees’ self-assessed achievement of competence and prioritization of relevant FOCs for residency exists [12], it is important to measure medical students’ self-assessment of relevant FOCs for residency before they finish their undergraduate training. These data are important to consider before considering potential changes in educational curricula. The aim of our study was to examine how a population of final-year undergraduate students assessed their own performance in the top ten facets of competence shortly before entering residency. We also compared students’ FOC self-assessment with the senior physicians’ ranking of relevance of these FOCs for beginning residents. We also analysed possible gender differences in these findings. We hypothesize that students’ self-assessment of competence will provide key insights into whether an achievement gap in competence exists at the transition from undergraduate to postgraduate medical education.

## Methods

### Study design and participants

As part of a large national survey targeting final-year medical students of a six-year undergraduate curriculum in Germany, an online questionnaire was developed using the software LimeSurvey (LimeSurvey GmbH, Hamburg, Germany). It took about 15 to 20 min to complete and contained 46 questions including a self-assessment in the top ten facets of competence required for entrustment decisions for beginning residents [10], questions about professional career planning including choice of medical specialty, a personality questionnaire, and sociodemographic data including age and gender. The link to the online questionnaire was sent to every German medical school (*N* = 37) in two tranches between October 2019 and July 2020 with the request of forwarding it to their final-year students (year six of the standard six-year undergraduate medical education). As incentive, participants could enter into a raffle of ten Apple iPads. Prior to voluntarily answering the questionnaire, participants provided informed consent electronically. All data was processed and analysed anonymously. The study was performed in accordance with the Declaration of Helsinki and the Local Psychological Ethics Committee at the Center for Psychosocial Medicine at the University Medical Center Hamburg-Eppendorf approved this study and confirmed its innocuousness (LPEK-0042). All participants gave their written consent and their participation was anonymized and voluntary.

### Instrument

#### Top ten competences for entrustment decisions in first year residents

In a Delphi study, 25 FOCs important for physicians were established [8] and ranked by Dutch and German medical educators with respect to their relevance for entrustment decisions in beginning residents [9]. The ranking scale was: 1: least important, 2: less important, 3: important, 4: very important, 5: most important [9]. This ranking study was then repeated with 202 German physicians (residents, consultants, supervising attendings, and department directors) from three medical faculties [10]. For the development of our questionnaire, we used the top ten FOCs for entrustment decisions in beginning residents [10]. Working with these FOC’s definitions [8] we then rephrased each FOC with respect to self-assessment, e.g., ‘Teamwork and collegiality’: “I cooperate effectively and respectfully in a (multidisciplinary) team and take the views, knowledge, and expertise of others into account”. We asked the participants to assess their own level of performance for each FOC individually on a 5-point Likert scale (1: strongly disagree, 2: disagree, 3: neither disagree nor agree, 4: agree, 5: strongly agree).

### Data processing

Data analysis was performed with IBM SPSS Statistics version 26 (IBM Corp., Armonk, N.Y., USA) and the general alpha-level was set to .005 to correct for multiple testing according to the Bonferroni method. To establish the FOCs’ order with respect to self-assessed performance level, means and standard deviations were calculated and within-group differences between adjacent performance levels were analysed with paired *t*-tests on a Bonferroni-corrected alpha-level of .006 as there were nine comparisons. The level of students’ self-assessed FOC performance was compared descriptively to the previously established ranking of FOC relevance for beginning residents by physicians [10]. Gender differences in self-assessed FOCs were analysed with independent *t*-tests.

## Results

A total of 1111 students from 35 medical faculties participated and 1083 (97.5%) completed the survey. This resembles an estimated response rate of 10% based on the annual number of medical graduates in Germany. Of the participating students, the mean age was 27.2 ± 3.1 years and 711 were female (65.8%), both representing percentages similar to all medical school graduates.

**Fig. 1:**
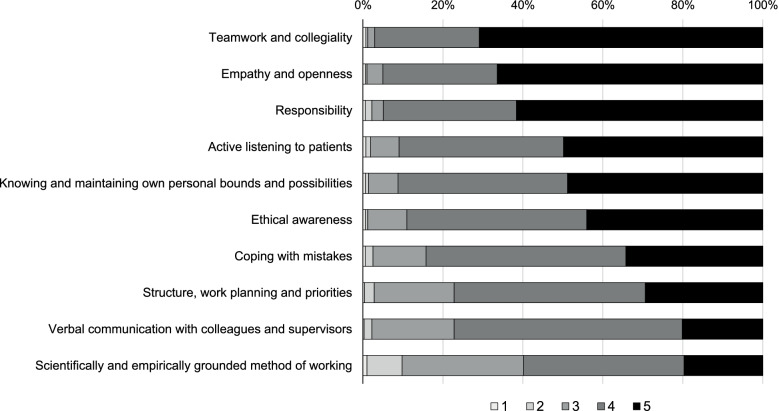
Percentage of Likert scale point ratings of the ten self-assessed facets of competence in order of self-assessed performance level (*n* = 1083)

Figure 1 shows the order of self-assessed competence on the ten FOCs according to the proportion of respective answers on the 5 point Likert scale. The majority of participants either chose 5 (‘strongly agree’) or 4 (‘agree’) regarding their performance on the ten FOCs. The percentage of lower scale ratings (‘strongly disagree’, ‘disagree’, and ‘neither agree nor disagree’) were most marked for these FOCs: ‘Scientifically and empirically grounded method of working’ (> 40%), ‘Structure, work planning, and priorities’ and ‘Verbal communication with colleagues and supervisors’ (both > 20%), ‘Ethical awareness’ and ‘Coping with mistakes’ (both > 10%), as compared to ‘Teamwork and collegiality’ (< 3%).

Comparing the level of the final-year students’ self-assessed performance of the FOCs and the relevance of these FOCs for beginning residents as rated by physicians [10] descriptively (Table 1), we found that the students’ self-assessed competence on the following FOCs was higher than the physicians’ ranking of these FOCs’ importance for beginning residents: ‘Teamwork and collegiality’, ‘Empathy and openness’, ‘Active listening to patients’, ‘Ethical awareness’, and ‘Verbal communication with colleagues and supervisors’. In contrast, these FOCs had lower student self-assessment of performance than the ranks of importance established by the physicians: ‘Responsibility’, ‘Knowing and maintaining own personal bounds and possibilities’, ‘Structure, work planning, and priorities’, ‘Coping with mistakes’, and ‘Scientifically and empirically grounded method of working’.

**Table 1:** Order of self-assessed performance level of the ten facets of competence (FOC) compared to physicians’ ranking of the competences’ importance for beginning residents

FOC	Final year students (***n*** = 1083)^**a**^	Physicians [10] (***n*** = 202)	Difference
Teamwork and collegiality	**1**	**3**	**+ 2**
Empathy and openness	**2**	**4**	**+ 2**
Responsibility	**3**	**1**	**−2**
Active listening to patients	**4**	**7**	**+ 3**
Knowing and maintaining own personal bounds and possibilities	**5**	**2**	**−3**
Ethical awareness	**6**	**9**	**+ 3**
Coping with mistakes	**7**	**6**	**−1**
Structure, work planning and priorities	**8**	**5**	**−3**
Verbal communication with colleagues and supervisors	**9**	**10**	**+ 1**
Scientifically and empirically grounded method of working	**10**	**8**	**−2**

^a^FOCs’ order of mean self-assessed level of performance: 1 being the FOC with the highest mean level, 10 being the FOC with the lowest mean level

On average (Table 2), final-year students rated their performance positively, with the FOCs reaching a mean > 4 (‘agree’) down to 8th place, ‘Structure, work planning and priorities’. The FOCs ‘Verbal communication with colleagues and supervisors’ and ‘Scientifically and empirically grounded method of working’ showed the lowest levels of performance self-assessment. Both of these differed significantly from ‘Teamwork and collegiality’ (1st place) with large effect sizes (‘Verbal communication with colleagues and supervisors’, *d* = 1.07, ‘Scientifically and empirically grounded method of working’, *d* = 1.25). All paired differences between two adjacent FOCs were significant, except for the FOCs on 2nd versus 3rd place, 4th versus 5th place, and 5th versus 6th place. Moderate effects (Cohen’s *d* > .5) could be detected for between self-assessed FOCs’ Δ means > .35 (SEM = .02), large effects (Cohen’s *d* > .8) for Δ means > .59 (SEM = .02).

**Table 2:**
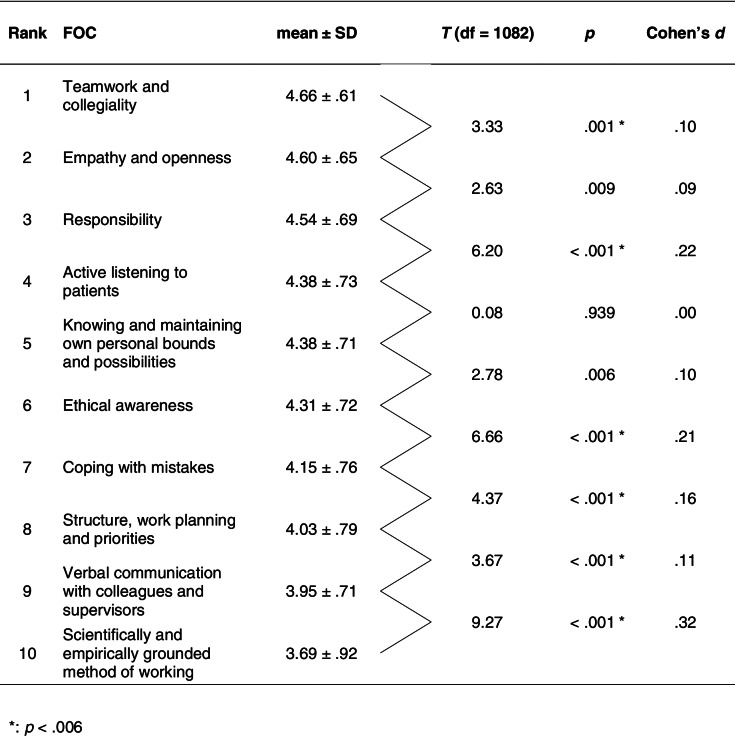
Order of self-assessed mean performance levels and within-group differences of the ten FOCs

The independent *t*-tests comparing mean female and male ratings of the FOCs yielded small but significant effects. Women rated their performance of the FOCs ‘Teamwork and collegiality’ (4.70 ± 0.57, *p* = .003, Cohen’s *d* = .20), ‘Empathy and openness’ (4.66 ± 0.59, *p* < .001, Cohen’s *d* = .29), and ‘Knowing and maintaining own personal bounds and possibilities’ (4.44 ± 0.66, *p* < .001, Cohen’s *d* = .27) significantly higher than men did (4.58 ± 0.68, 4.48 ± 0.73, and 4.25 ± 0.78, respectively), while men showed higher self-assessed performance in the FOC ‘Scientifically and empirically grounded method of working’ (3.91 ± 0.92, *p* < .001, Cohen’s *d* = .38) than women did (3.57 ± 0.89).

## Discussion

In this study, we focused on final-year medical students’ self-assessment of facets of competence (FOC) at the time of their transition from undergraduate to postgraduate medical training. The ten FOCs of relevance for beginning residents were presented according to students’ self-assessed mastery of each FOCs. Significant differences between the mean levels of self-assessed FOCs were observed. ‘Teamwork and collegiality’ reached the highest self-assessed performance which may reflect the content of undergraduate medical curricula where teamwork is taught as a basic requirement for good patient care [20–22]. The final-year students assessed themselves lowest in ‘Verbal communication with colleagues and supervisors’ and ‘Scientifically and empirically grounded method of working’, suggesting the need for improving these FOCs during undergraduate medical education. Communication skills training is offered in most medical school programmes [23], but still mostly focuses on communicating with patients and rarely with colleagues or supervisors [24]. However, interprofessional communication has already become part of a European consensus statement on core communication skills in the health professions [25]. Such training has been implemented, for instance, on an interprofessional training ward for undergraduate medical students [26, 27] and can also be accomplished in interprofessional simulations [28]. The low performance self-assessment of ‘Scientifically and empirically grounded method of working’ could be due to a lack of clinical reasoning training in the undergraduate medical curricula [29]. More exercises in evidence-based medicine, which is a prerequisite for clinical decision making [30, 31] as well as in clinical reasoning [32] could also improve medical graduates’ preparedness for residency, in which ‘Empathy and openness’, which was highly rated, must be combined with a ‘Scientifically and empirically grounded method of working’ [33].

With respect to the relevance of the FOCs for beginning residents [10], physicians ranked ‘Responsibility’, ‘Knowing and maintaining own personal bounds and possibilities’, ‘Structure, work planning and priorities’, ‘Coping with mistakes’, and ‘Scientifically and empirically grounded method of working’ higher than final-year students self-assessed their performance on the respective FOC. This could also be a hint that these FOCs, which are relevant for successful transition from undergraduate to postgraduate training, should receive more attention in the undergraduate curriculum. For FOCs like ‘Responsibility’, ‘Knowing and maintaining own personal bounds and possibilities’, and ‘Coping with mistakes’, all a part of medical professionalism, additional training programmes and general principles have been suggested [34, 35].

The FOC ‘Structure, work planning and priorities’ includes management skills and multitasking, which are required in physicians’ daily work and caused strain in medical students when they participated in the physician’s role in a simulated first day of residency [36]. Therefore, management skills are recommended to be incorporated as learning objectives in undergraduate medical curricula [37]. Management skills are part of the health system sciences which should complement the basic and clinical sciences. They lead to full integration of all health system-related competences needed in medical education for graduates to provide for comprehensive patient care [38]. Multitasking simulations can help medical trainees learn how to handle demanding management tasks and potentially reduce the stress accompanying work planning and prioritizing [39]. However, special training for such health system-related competences could also be included at the beginning of postgraduate training [40].

In our study, female students assessed themselves higher in ‘Empathy and openness’, ‘Teamwork and collegiality’, and ‘Knowing and maintaining own personal bounds and possibilities’ than male students did. In other studies, female medical students also scored higher in self-evaluation of empathy [41], felt more confident in their ability to listen and to work as part of a team [42], and received higher scores for teamwork and knowing personal bounds in a workplace-based assessment [12]. Male students, in our study, assessed themselves more competent in ‘Scientifically and empirically grounded method of working’ than did female students, which has been demonstrated before in self-assessment [43]. However, it should be noted that this FOC showed the lowest mean level for both genders. Female students’ lower self-assessment with respect to scientifically based work could be due to their identity formation confirming to traditional roles [44] or due to teachers treating female students according to these roles [44]. In general, females often underestimate their performances, while men tend to overestimate them [15, 45]. Medical schools need to be aware of such gender differences and could consider providing gender-specific mentoring programmes [46] to reduce these gender-specific differences.

As to study limitations, the nature of our online study might have led to a self-selection bias, as more interested and motivated final-year students chose to participate. This bias might also have resulted in overconfident self-assessments of competence. The questionnaire’s wording could have led to a social desirability bias. Our sample consisted of twice as many women as men. This roughly resembles the current distribution among medical students [47], but the differences in self-assessment with respect to gender show that the results could be confounded by the overrepresentation of women. A weak point in the study design is that the pre-established ranking by physicians [10] and the self-assessed FOC performance levels from this present study are not statistically comparable, but can only descriptively define areas for improvement. Additionally, medical students’ self-assessment often is inaccurate [15], which hampers the questionnaire’s reliability. Since we do not know the exact number of students reached at the included medical faculties, we conservatively estimated a response rate of only 10%. Nevertheless, a strength of our study is the large study sample with over 1000 participants, which enhances the power of the analysis in favour of generalizability. Our national online survey was designed so it could prospectively follow participants during their medical careers. This could include regular self-assessment of competences over the course of the final-year and during later stages of residency. In addition to self-assessment, students’ supervisors should also rate the trainees’ competences in order to monitor the development of these competences and to provide feedback to support the transition from undergraduate to postgraduate training. Our findings emphasise that health system-related competences should become a more prominent learning objective in undergraduate medical education, in order to ease students’ transition to postgraduate training.

## Conclusions

Self-assessment of facets of competence by final-year medical students in a national survey revealed important insights into the transition from undergraduate to postgraduate training. The undergraduate students rated themselves highest in ‘Teamwork and collegiality’ and ‘Empathy and openness’ while the lowest ratings were found for ‘Verbal communication with colleagues and supervisors’ and ‘Scientifically and empirically grounded method of working’. With respect to the importance of the facets of competence for beginning residents as ranked by physicians, competence areas related to health system sciences were found to need improvement during undergraduate training. These competences included the development of professionalism, management skills, or gender-specific learning opportunities. Self-assessment of competences may provide a useful instrument for monitoring the development of competences in medical training.

## Supplementary Information

**Additional file 1.**

## Data Availability

All data and materials are available from the corresponding author upon request.

## Abbreviation

FOC: Facet of competence

## References

[1] Harris P, Snell L, Talbot M, Harden RM (2010). Competency-based medical education: implications for undergraduate programs. Med Teach.

[2] Albanese MA, Mejicano G, Anderson WM, Gruppen L (2010). Building a competency-based curriculum: the agony and the ecstasy. Adv Health Sci Educ Theory Pract.

[3] Frank JR, Snell L, Sherbino J (2015). CanMEDS 2015 physician competency framework.

[4] Fischer MR, Bauer D, Mohn K, NKLM-Projektgruppe. Finally finished! National competence based catalogues of learning objectives for undergraduate medical education (NKLM) and dental education (NKLZ) ready for trial. GMS. J Med Educ. 2015;32(3):Doc35. 10.3205/zma000977 PMC4580444 26677513

[5] Wijnen-Meijer M, van der Schaaf M, Booij E, Harendza S, Boscardin C, van Wijngaarden J, Ten Cate TJ (2013). An argument-based approach to the validation of UHTRUST: can we measure how recent graduates can be trusted with unfamiliar tasks?. Adv Health Sci Educ Theory Pract..

[6] Prediger S, Schick K, Fincke F, Fürstenberg S, Oubaid V, Kadmon M, Berberat PO, Harendza S (2020). Validation of a competence-based assessment of medical students’ performance in the physician’s role. BMC Med Educ..

[7] Iobst WF, Sherbino J, Ten Cate O, Richardson DL, Dath D, Swing SR, Harris P, Mungroo R, Holmboe ES (2010). Frank JR for the international CBME collaborators. Competency-based medical education in postgraduate medical education. Med Teach..

[8] Wijnen-Meijer M, van der Schaaf M, Nillesen K, Harendza S, Ten Cate O (2013). Essential facets of competence that enable trust in graduates: a Delphi study among physician educators in the Netherlands. J Grad Med Educ..

[9] Wijnen-Meijer M, van der Schaaf M, Nillesen K, Harendza S, Ten Cate O (2013). Essential facets of competence that enable trust in medical graduates: a ranking study among physician educators in two countries. Perspect Med Educ.

[10] Fürstenberg S, Schick K, Deppermann J, Prediger S, Berberat PO, Kadmon M, Harendza S (2017). Competencies for first year residents – physicians’ views from medical schools with different undergraduate curricula. BMC Med Educ..

[11] Fürstenberg S, Harendza S (2017). Differences between medical student and faculty perceptions of the competencies needed for the first year of residency. BMC Med Educ..

[12] Fincke F, Prediger S, Schick K, Fürstenberg S, Spychala N, Berberat PO, Harendza S, Kadmon M (2020). Entrustable professional activities and facets of competence in a simulated workplace-based assessment for advanced medical students. Med Teach..

[13] Leach DC (2002). Competence is a habit. JAMA..

[14] Duffy FD, Holmboe ES (2006). Self-assessment in lifelong learning and improving performance in practice: physician know thyself. JAMA..

[15] Blanch-Hartigan D (2011). Medical students’ self-assessment of performance: results from three meta-analyses. Patient Educ Couns.

[16] Epstein RM (2007). Assessment in medical education. N Engl J Med.

[17] Ochsmann EB, Zier U, Drexler H, Schmid K (2011). Well prepared for work? Junior doctors' self-assessment after medical education. BMC Med Educ..

[18] Minter RM, Amos KD, Bentz ML, Blair PG, Brandt C, D’Cunha J, Davis E, Delman KA, Deutsch ES, Divino C (2015). Transition to surgical residency: a multi-institutional study of perceived intern preparedness and the effect of a formal residency preparatory course in the fourth year of medical school. Acad Med.

[19] Franzen D, Kost A, Knight C (2015). Mind the gap: the bumpy transition from medical school to residency. J Grad Med Educ.

[20] Freytag J, Stroben F, Hautz WE, Eisenmann D, Kämmer JE (2017). Improving patient safety through better teamwork: how effective are different methods of simulation debriefing? Protocol for a pragmatic, prospective and randomised study. BMJ Open.

[21] Escher C, Creutzfeldt J, Meurling L, Hedman L, Kjellin A, Felländer-Tsai L (2017). Medical students’ situational motivation to participate in simulation based team training is predicted by attitudes to patient safety. BMC Med Educ.

[22] Chakraborti C, Boonyasai RT, Wright SM, Kern DE (2008). A systematic review of teamwork training interventions in medical student and resident education. J Gen Intern Med.

[23] Smith S, Hanson JL, Tewksbury LR, Christy C, Talib NJ, Harris MA, Beck GL, Wolf FM (2007). Teaching patient communication skills to medical students: a review of randomized controlled trials. Eval Health Prof.

[24] Kessler CS, Chan T, Loeb JM, Malka ST (2013). I’m clear, you’re clear, we’re all clear: improving consultation communication skills in undergraduate medical education. Acad Med.

[25] Bachmann C, Abramovitch H, Barbu CG, Cavaco AM, Elorza RD, Haak R, Loureiro E, Ratajska A, Silverman J, Winterburn S, Rosenbaum M (2013). A European consensus on learning objectives for a core communication curriculum in health care professions. Patient Educ Couns.

[26] Mette M, Baur C, Hinrichs J, Narciß E (2021). Gaining interprofessional knowledge and interprofessional competence on a training ward. Med Teach..

[27] Oosterom N, Floren L, ten Cate O, Westerveld H (2019). A review of interprofessional training wards: enhancing student learning and patient outcomes. Med Teach..

[28] Ross AJ, Anderson JE, Kodate N, Thomas L, Thompson K, Thomas B, Key S, Jensen H, Schiff R, Jaye P (2013). Simulation training for improving the quality of care for older people: an independent evaluation of an innovative programme for inter-professional education. BMJ Qual Saf.

[29] Kononowicz AA, Hege I, Edelbring S, Sobocan M, Huwendiek S, Durning SJ (2020). The need for longitudinal clinical reasoning teaching and assessment: results of an international survey. Med Teach..

[30] Hecht L, Buhse S, Meyer G (2016). Effectiveness of training in evidence-based medicine skills for healthcare professionals: a systematic review. BMC Med Educ..

[31] Maggio LA, Tannery NH, Chen HC, Ten Cate O, O’Brien B (2013). Evidence-based medicine training in undergraduate medical education: a review and critique of the literature published 2006-2011. Acad Med.

[32] Harendza S, Krenz I, Klinge A, Wendt U, Janneck M (2017). Implementation of a clinical reasoning course in the internal medicine trimester of the final year of undergraduate medical training and its effect on students' case presentation and differential diagnostic skills. GMS J Med Educ.

[33] Aper L, Veldhuijzen W, Dornan T, van de Ridder M, Koole S, Derese A, et al. "should I prioritize medical problem solving or attentive listening?": the dilemmas and challenges that medical students experience when learning to conduct consultations. Patient Educ Couns. 2015;98(1):77–84. 10.1016/j.pec.2014.09.016 25448312

[34] Birden H, Glass N, Wilson I, Harrison M, Usherwood T, Nass D (2013). Teaching professionalism in medical education: a best evidence medical education (BEME) systematic review. BEME guide no. 25. Med Teach..

[35] Cruess RL, Cruess SR (2006). Teaching professionalism: general principles. Med Teach..

[36] Fürstenberg S, Prediger S, Kadmon M, Berberat PO, Harendza S (2018). Perceived strain of undergraduate medical students during a simulated first day of residency. BMC Med Educ..

[37] Myers CG, Pronovost PJ (2017). Making management skills a core component of medical education. Acad Med.

[38] Gonzalo JD, Chang A, Dekhtyar M, Starr SR, Holmboe E, Wolpaw DR (2020). Health systems science in medical education: unifying the components to catalyze transformation. Acad Med.

[39] Adams TN, Rho JC (2017). Multitasking simulation: present application and future directions. Med Teach..

[40] Wong BM, Holmboe ES (2016). Transforming the academic faculty perspective in graduate medical education to better align educational and clinical outcomes. Acad Med.

[41] Hojat M, Gonnella JS, Mangione S, Nasca TJ, Veloski JJ, Erdmann JB, Callahan CA, Magee M (2002). Empathy in medical students as related to academic performance, clinical competence and gender. Med Educ.

[42] Clack GB, Head JO (1999). Gender differences in medical graduates' assessment of their personal attributes. Med Educ.

[43] Bakken LL, Sheridan J, Carnes M (2003). Gender differences among physician–scientists in self-assessed abilities to perform clinical research. Acad Med.

[44] Lurie SJ, Meldrum S, Nofziger AC, Sillin LF, Mooney CJ, Epstein RM (2007). Changes in self-perceived abilities among male and female medical students after the first year of clinical training. Med Teach..

[45] Cooney CM, Aravind P, Lifchez SD, Hultman CS, Weber RA, Brooke S, Cooney DS (2021). Differences in operative self-assessment between male and female plastic surgery residents: a survey of 8,149 cases. Am J Surg.

[46] House A, Dracup N, Burkinshaw P, Ward V, Bryant LD (2021). Mentoring as an intervention to promote gender equality in academic medicine: a systematic review. BMJ Open.

[47] Ziegler S, Zimmermann T, Krause-Solberg L, Scherer M, van den Bussche H. Male and female residents in postgraduate medical education – a gender comparative analysis of differences in career perspectives and their conditions in Germany. GMS. J Med Educ. 2017;34(5):Doc53. 10.3205/zma001130 PMC5704604 29226221

