# Supplementary material for: Final-year medical students’ self-assessment of facets of competence for beginning residents
Source: BMC Med Educ. 2022 Feb 7;22:82. doi: 10.1186/s12909-021-03039-2 (PMC8822672; doi:10.1186/s12909-021-03039-2)
Supplement: Supplementary file 1 — Additional file 1. [file 12909_2021_3039_MOESM1_ESM.docx]

# Final-year medical students’ self-assessment of facets of competence for beginning residents

**Lisa Bußenius^1^, Sigrid Harendza^2^, Hendrik van den Bussche^3^, Susan Selch^1,3^**

^1^Department of Biochemistry and Molecular Cell Biology, Center for Experimental Medicine, University Medical Center Hamburg-Eppendorf, Germany

^2^III. Department of Internal Medicine, University Medical Center Hamburg-Eppendorf, Germany

^3^Department of General Practice and Primary Care, University Medical Center Hamburg-Eppendorf, Germany

**Facets of competence questionnaire**

Below you will find 10 medical competences. Please assess to what extent the statements currently apply to you.

|  | strongly disagree | disagree | neither agree  nor disagree | agree | strongly agree |
| --- | --- | --- | --- | --- | --- |
| **Responsibility**  I take responsibility and show accountability for my work. | ◯ | ◯ | ◯ | ◯ | ◯ |
| **Teamwork and collegiality**  I cooperate effectively and respectfully in a (multidisciplinary) team, taking the views, knowledge, and expertise of others into account. | ◯ | ◯ | ◯ | ◯ | ◯ |
| **Empathy and openness**  I show empathy, openness, and susceptibility/accessibility in my contact with patients. | ◯ | ◯ | ◯ | ◯ | ◯ |
| **Knowing and maintaining own personal bounds and possibilities**  I know the boundaries of my own ability and ask for help (timely) when needed. I reflect on myself and the situation. | ◯ | ◯ | ◯ | ◯ | ◯ |
| **Verbal communication with colleagues and supervisors**  I give structured, pithy, and unambiguous verbal reports on my findings on a patient and my diagnostic and therapeutic policy. I ask relevant and purposeful questions. | ◯ | ◯ | ◯ | ◯ | ◯ |
| **Structure, work planning and priorities**  I see the overall picture, have organizational skills and a flexible attitude, and set priorities in my work. | ◯ | ◯ | ◯ | ◯ | ◯ |
| **Empirically and scientifically grounded method of working**  I use evidence-based procedures whenever possible and rely on scientific knowledge. I search actively and purposefully for evidence and consult high-quality resources. I use my scientific knowledge critically and carefully in my work. | ◯ | ◯ | ◯ | ◯ | ◯ |
| **Active listening to patients**  I listen actively to patients and react (verbally and nonverbally) to the things I hear in a way that encourages the sharing of information (by the patients) and confirms my involvement with the patient. I show attention to nonverbal signals coming from the patients. | ◯ | ◯ | ◯ | ◯ | ◯ |
| **Coping with mistakes**  I am aware of the fact that anyone can make and does make mistakes once in a while. I am approachable when someone points out my mistakes and react adequately when I think that a colleague makes a mistake. | ◯ | ◯ | ◯ | ◯ | ◯ |
| **Ethical awareness**  I am acquainted with ethical aspects of my work. I distinguish different points of view in the moral debate and make deliberate choices when my work confronts me with ethical issues. | ◯ | ◯ | ◯ | ◯ | ◯ |
